# Synergistic anti-tumor effect of bullfrog sialic acid-binding lectin and pemetrexed in malignant mesothelioma

**DOI:** 10.18632/oncotarget.17198

**Published:** 2017-04-18

**Authors:** Toshiyuki Satoh, Takeo Tatsuta, Shigeki Sugawara, Akiyoshi Hara, Masahiro Hosono

**Affiliations:** ^1^ Department of Clinical Pharmacotherapeutics, Tohoku Medical and Pharmaceutical University, Aobaku, Sendai, Miyagi 981-8558, Japan; ^2^ Division of Cell Recognition Study, Institute of Molecular Biomembrane and Glycobiology, Tohoku Medical and Pharmaceutical University, Aobaku, Sendai, Miyagi 981-8558, Japan

**Keywords:** malignant mesothelioma, apoptosis, cell cycle, sialic acid-binding lectin, combination treatment

## Abstract

Malignant mesothelioma is an aggressive cancer with limited therapeutic options. Sialic acid-binding lectin isolated from *Rana catesbeiana* oocytes (cSBL) is a multifunctional protein with anti-cancer activity. The effects of pemetrexed, cisplatin, and cSBL were evaluated in mesothelioma and normal mesothelial cell lines. We evaluated cytotoxicity, apoptosis, caspase-3 cleavage and activation, cell proliferation, cell cycle arrest, and levels of cell cycle proteins in H28 cells treated with pemetrexed, cisplatin, and cSBL alone or in combination. Treatment with cSBL alone was cytotoxic to mesothelioma cells. The anti-cancer effect of cSBL was observed in a broader range of cell lines and exhibited greater cancer cell selectivity than pemetrexed or cisplatin. Combination treatment with pemetrexed + cSBL resulted in greater dose-dependent cytotoxicity than pemetrexed + cisplatin, the standard of care in mesothelioma. The synergistic effect of pemetrexed + cSBL was mediated by the cytostatic effect of pemetrexed and the cytotoxic effect of cSBL. It thus appears that cSBL has therapeutic potential for the treatment of mesothelioma.

## INTRODUCTION

Malignant mesothelioma is an aggressive cancer of mesothelial cell origin that results from exposure to asbestos [1, 2]. Asbestos was extensively used in industry and construction during the 20th century. It was first associated with the incidence of mesothelioma in the 1960s [3–6]. Because mesothelioma develops 20–30 years after asbestos exposure, the number of mesothelioma patients is expected to increase [7–9]. There are few therapeutic options for mesothelioma. The folate antimetabolite pemetrexed is a chemotherapeutic that is typically used in combination with platinum-containing drugs such as cisplatin [10, 11]. Compared to cisplatin monotherapy, combination treatment with pemetrexed + cisplatin improves response rate, progression-free survival, overall survival, and quality of life in mesothelioma patients [10]. However, most patients treated with pemetrexed and cisplatin experience tumor progression or relapse within a year [12, 13]. Drug resistance is also commonly observed [14]. Therefore, alternative therapeutic agents for mesothelioma are needed.

Sialic acid-binding lectin isolated from *Rana catesbeiana* oocytes (cSBL) is a multifunctional protein with lectin-binding [15, 16], ribonuclease (RNase) [17], and anti-tumor activity [16]. cSBL is cytotoxic to cancer cells including leukemia [18–21], breast carcinoma [21–24], mesothelioma [25], and hepatoma cells [21, 26, 27]. It has little effect on normal cells such as fibroblasts, melanocytes, keratinocytes, and mesothelial cells [20, 21, 25, 26, 28]. cSBL-induced cell death involves at least three steps: (1) binding to the cell surface via carbohydrate chain containing sialic acid, (2) cell internalization, and (3) RNA cleavage and activation of apoptosis. The cytotoxic effects of cSBL are mediated by the induction of apoptosis in response to mitochondrial perturbation. RNase activity is essential for cSBL-induced cytotoxicity [24]. Treatment of tumor-bearing mice (transplanted with sarcoma 180 cells, Ehrlich, or Mep 2 ascites cells) with cSBL at a non-toxic dose prolonged survival [16]. In contrast to commonly used DNA-targeting agents, the cytotoxic effects of RNases are non-genotoxic [29]. Thus, cSBL has therapeutic potential as a novel RNA-targeting anti-cancer agent.

Combination chemotherapy is the standard of care for many cancers. It allows for the use of doses that maximize the therapeutic effects while preventing chemoresistance. cSBL has an anti-cancer effect in mesothelioma cell lines (e.g. NCI-H28 [H28], ACC-MESO-1 [MESO-1], and ACC-MESO-4 [MESO-4]), and exhibited synergistic effects with tumor necrosis factor-related apoptosis-inducing ligand (TRAIL) in H28 cells [25] and interferon-γ in hepatoma cell lines [27]. We investigated whether cSBL exhibited greater tumor selectivity than pemetrexed and cisplatin, and whether combination treatment with cSBL + pemetrexed was comparable or superior to combination treatment with pemetrexed + cisplatin.

## RESULTS

### cSBL exhibits greater cancer cell selectivity than pemetrexed and cisplatin

**Figure 1:**
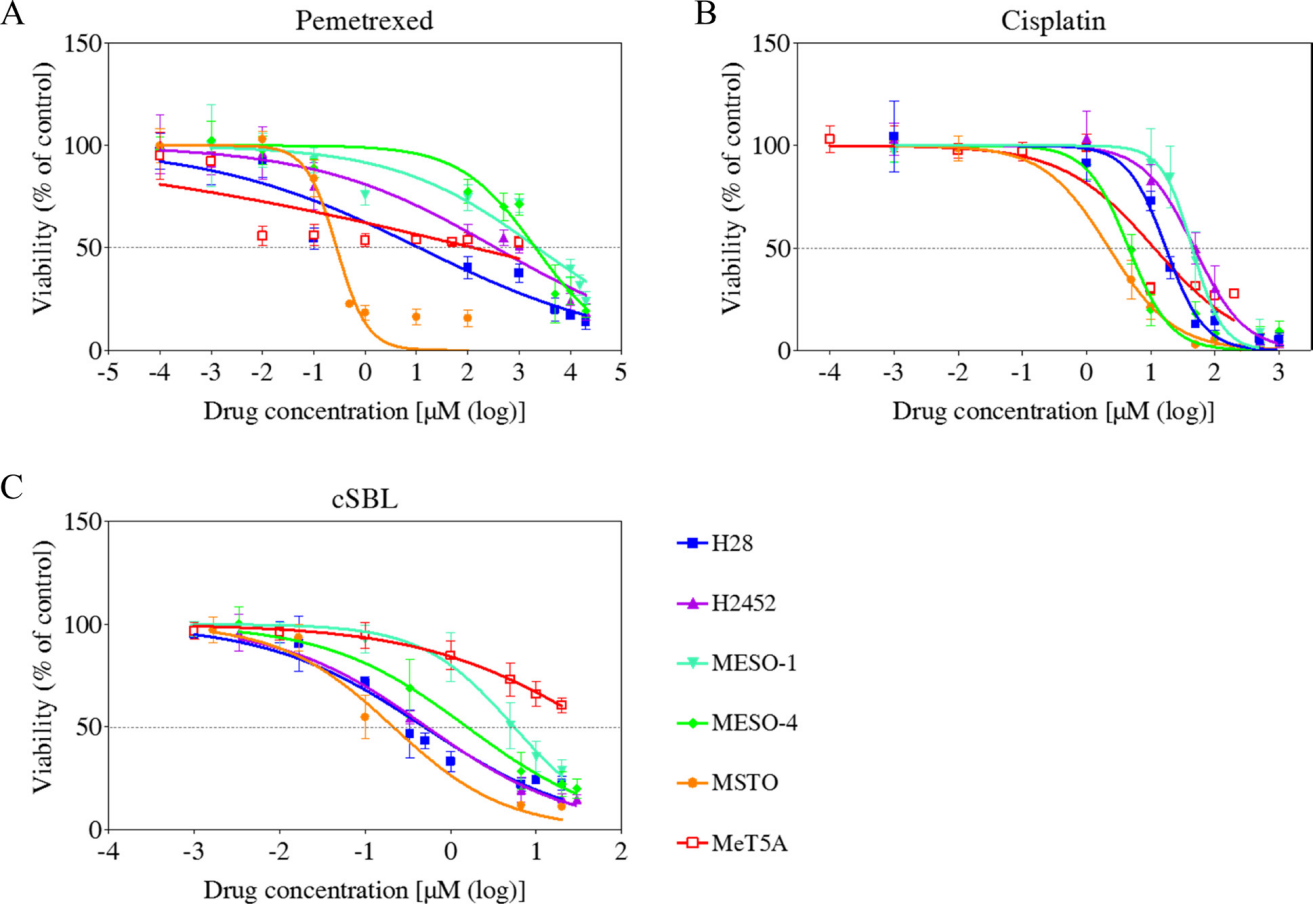
Dose-response curves in the mesothelioma cell lines (H28, H2452, MESO-1, MESO-4, and MSTO), and MeT5A mesothelial cells treated with pemetrexed (**A**), cisplatin (**B**), or cSBL (**C**). Cells were treated with pemetrexed (0.1 nM–20 mM), cisplatin (1 nM–1 mM), or cSBL (1 nM–30 μM) for 72 h. The dots and bars represent the mean and SD, respectively. Dose-response curves are depicted as lines or dotted lines. Each data point represents the mean ± SD of at least three independent WST-8 assays. Each sample was plated in triplicate.

We evaluated the effects of cSBL, pemetrexed, and cisplatin on the viability of epithelioid mesothelioma cells (NCI-H2452 [H2452], MESO-1, and MESO-4), biphasic mesothelioma cells MSTO-211H (MSTO) and sarcomatoid mesothelioma cells (H28), and non-malignant mesothelial cells (MeT5A) using WST-8 assays. All three agents reduced mesothelioma cell viability. However, cSBL had the least effect on MeT5A cells (Figure 1). Even at the highest concentration (20 μM), cSBL only inhibited MeT5A cell viability by 40% (Figure 1C). In contrast, pemetrexed decreased Met5A cell viability by 50% at 0.01 μM and cisplatin decreased viability by 70% at 10 μM. We calculated the half maximal inhibitory concentration (IC_50_), defined as the concentration required to inhibit cell growth by 50%, from dose-response curves. The relative sensitivity (RS) of each agent represents the ratio of the IC_50_ value in a cancer cell line to the IC_50_ value in MeT5A cells (Table 1). H2452, MESO-1, and MESO-4 cells were resistant to pemetrexed (RS: 0.37, 0.06, and 0.06, respectively), and H28, H2452, and MESO-1 cells were resistant to cisplatin (RS: 0.66, 0.24, and 0.26, respectively). In contrast, cSBL was cytotoxic in these drug-resistant cell lines. The RS of cSBL was higher (9.48–247.02) than the RS values of pemetrexed and cisplatin in mesothelioma cells, indicating that the cytotoxic effect of cSBL was more selective to cancer cells.

**Table 1:** IC_50_ values (μM) and RS of pemetrexed, cisplatin, and cSBL in mesothelioma cells

Drugs	Drug targets		MeT5A	H28	H2452	MESO-1	MESO-4	MSTO
Pemetrexed	TS	IC_50_	129.50	11.27	353.00	2267.00	2077.00	0.28
	DHFR		(24.20–693.20)	(5.67–22.39)	(208.6- 597.3)	(1393–3691)	(1634–2639)	(0.23–0.34)
	GRAFT	RS	1.00	11.49	0.37	0.06	0.06	465.99
Cisplatin	DNA	IC_50_	11.27	17.18	47.62	44.14	4.54	2.23
			(8.07–15.73)	(15.14–19.50)	(41.23–55.00)	(38.62–50.46)	(3.87–5.33)	(1.62–3.06)
		RS	1.00	0.66	0.24	0.26	2.48	5.06
cSBL	RNA	IC_50_	52.22	0.46	0.52	5.51	1.54	0.21
			(33.94–80.36)	(0.35–0.68)	(0.41–0.66)	(4.67–6.50.)	(1.10–2.17)	(0.15–0.29)
		RS	1.00	113.89	100.00	9.48	33.91	247.02

The 95% confidence intervals for each IC_50_ value are shown in parentheses. The RS value was calculated as the IC_50_ value of each agent in MeT5A cells divided by the IC_50_ value in each cancer cell line.

### cSBL and pemetrexed exert a strong synergistic effect

**Figure 2:**
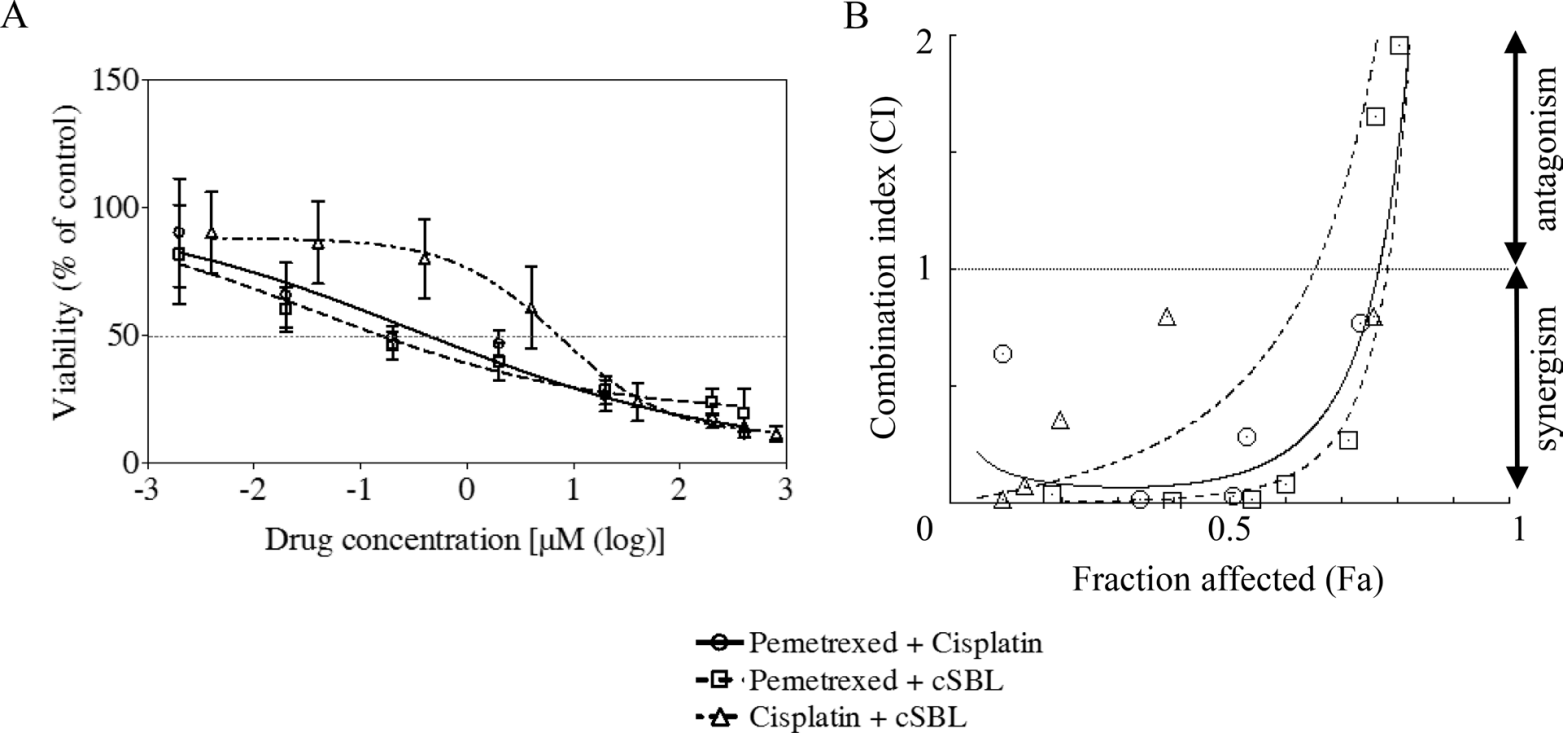
Pharmacological interactions between pemetrexed, cisplatin, and cSBL in H28 cells (**A**) The drug concentration ratios were as follows: pemetrexed + cisplatin (1:2), pemetrexed + cSBL (20:1), cisplatin + cSBL (40:1). Cells were treated with pemetrexed (2 nM–400 μM), cisplatin (4 nM–800 μM), or cSBL (0.1 nM–20 μM) for 72 h. The horizontal axis indicates the concentration of pemetrexed in the pemetrexed + cisplatin or pemetrexed + cSBL combination or the concentration of cisplatin in the cisplatin + cSBL combination. (**B**) CI-Fa curves for H28 cells treated with pemetrexed + cisplatin, pemetrexed + cSBL, or cisplatin + cSBL. CI values < 1 indicate a synergistic effect, and CI values > 1 indicate an antagonistic effect. Each data point represents the mean ± SD of three independent WST-8 assays. Each sample was plated in triplicate.

We investigated the pharmacological interaction between the three agents by evaluating the viability of H28 cells treated with pemetrexed + cisplatin, pemetrexed + cSBL, or cisplatin + cSBL. H28 cells are moderately sensitive to pemetrexed (Figure 1 and Table 1). We previously demonstrated that combination treatment with cSBL + TRAIL has a synergistic effect H28 cells [25]. The concentration of each drug in the combination regimen was based on the IC_50_ value of each agent as a single treatment. Pemetrexed + cisplatin and pemetrexed + cSBL reduced cell viability to a similar extent (Figure 2A). To evaluate the synergistic effect of each drug combination, we calculated combination index (CI) values. The CI curves shown in Figure 2B indicated that pemetrexed + cSBL had a stronger synergistic effect and broader fraction affected (Fa) range than the other combinations. Cisplatin + cSBL exhibited the weakest cytotoxic and synergistic effects. We also calculated the concentration of each agent at Fa = 0.5 (i.e., the concentration predicted to reduce cell viability by 50%) (Table 2). Lower concentrations of each agent were required to inhibit cell viability by 50% when they were combined rather than administered a single agents. The concentration of pemetrexed decreased by nearly 50% when used in combination with cSBL (0.38 μM) compared to cisplatin (0.65 μM).

**Table 2:** CI values and drug concentrations at Fa = 0.5 in H28 cells

	Drug/Combo	CI value	Concentration at Fa = 0.5		
			Pemetrexed (μM)	Cisplatin (μM)	cSBL (μM)
Single	Pemetrexed	−	20.44	−	−
	Cisplatin	−	−	15.2	−
	cSBL	−	−	−	0.69
Combination	Pemetrexed + Cisplatin	0.12	0.65	1.31	−
	Pemetrexed + cSBL	0.05	0.38	−	0.02
	Cisplatin + cSBL	0.47	−	4.6	0.12

### Pemetrexed and cSBL induce apoptosis in mesothelioma cells

**Figure 3:**
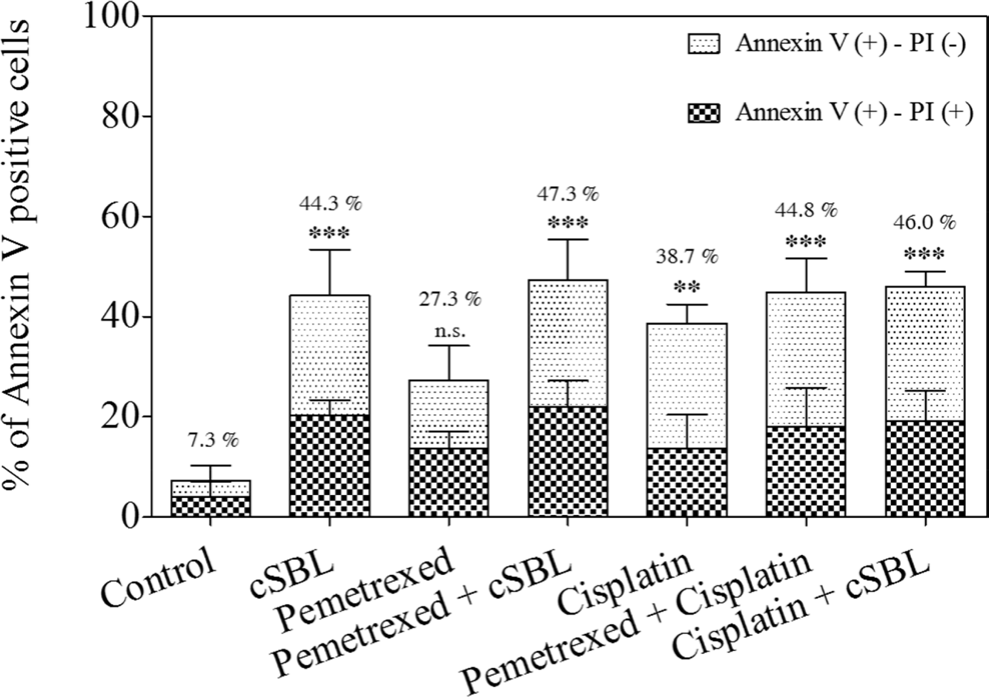
Pemetrexed, cisplatin, and cSBL, either alone or in combination, induced apoptosis in H28 cells Cells were treated with pemetrexed (20 μM), cisplatin (40 μM), or cSBL (1 μM) for 72 h. The y-axis indicates the percentage of annexin V-positive cells. The percentage of PI-positive and negative cells is indicated by the different column patterns. The statistical significance of the percentage of annexin V-positive cells compared to the control is shown. **p < 0.01, ***p < 0.001; n.s.: not significant.

We previously demonstrated that cSBL induces apoptosis in H28 (sarcomatoid histological type) as well as MESO-1 and MESO-4 (epithelioid type) cells, and that the synergistic anti-tumor effect of cSBL + TRAIL in H28 cells was mediated by an increase in apoptosis [25]. To elucidate the mechanism underlying the synergistic effect of pemetrexed + cSBL in H28 cells, we evaluated markers of apoptosis. In the initial combination treatment experiments (Figure 2A), pemetrexed (20 μM), cisplatin (40 μM), and cSBL (1 μM) reduced the viability of H28 cell lines to similar levels (approximately 30%). Therefore, we used these concentrations in all subsequent experiments. After 72 h of treatment, the percentage of annexin V-positive cells was 27.3%, 38.7%, and 44.3% in cells treated with pemetrexed, cisplatin, and cSBL, respectively, and 44.8%, 47.3%, and 46.0% in cells treated with pemetrexed + cisplatin, pemetrexed + cSBL, and cisplatin + cSBL, respectively (Figure 3). There were no statistically significant differences between the individual and combination treatments.

### The synergistic effect of pemetrexed + cSBL is not mediated by changes in caspase-3 activity

**Figure 4:**
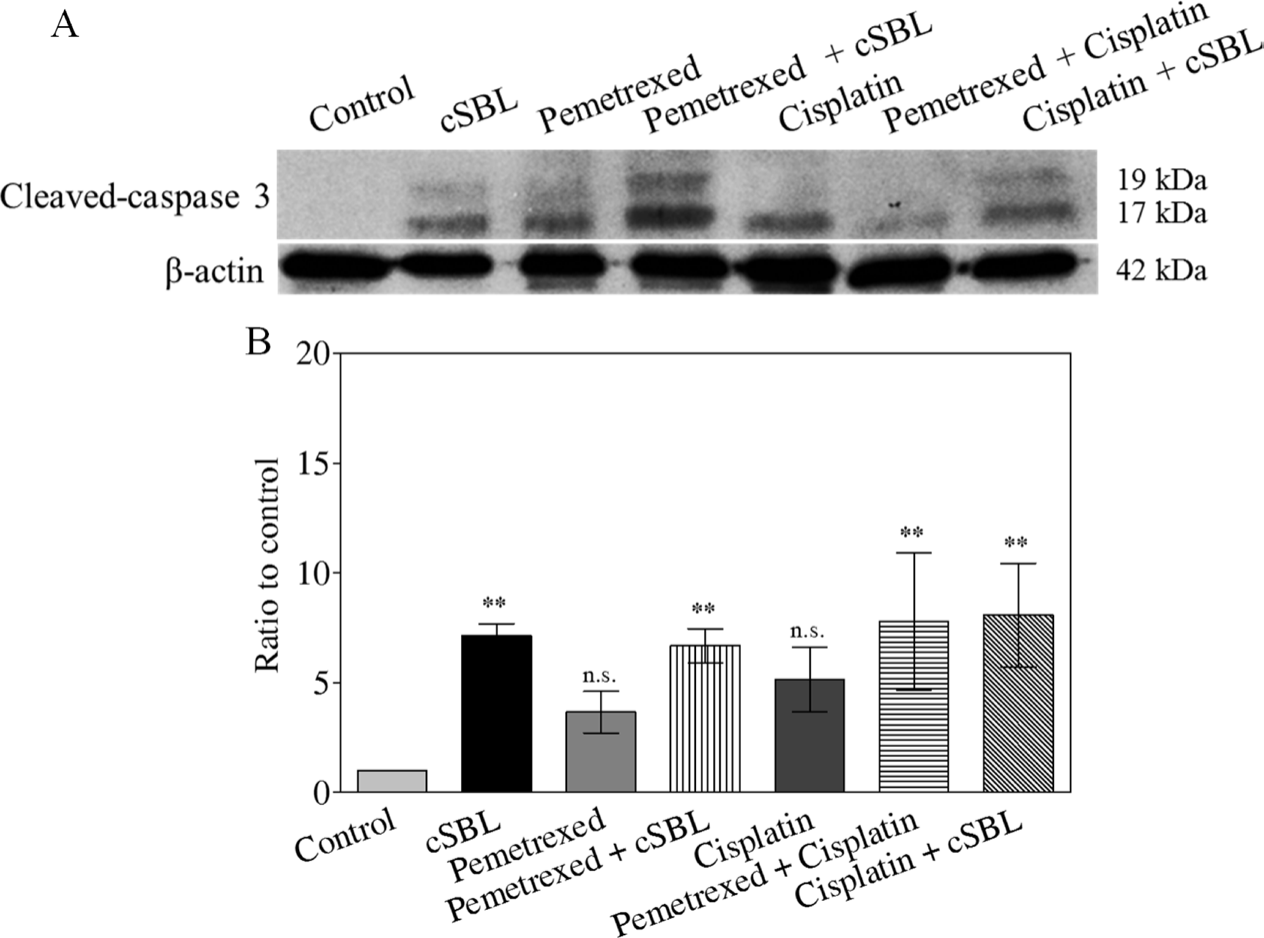
Caspase-3 activation is not enhanced by combination treatment Cells were treated with pemetrexed (20 μM), cisplatin (40 μM), or cSBL (1 μM) for 72 h. (**A**) Cleaved (activated) caspase-3 was detected using western blot analysis. (**B**) Caspase-3 activity was analyzed using a Caspase-Glo^™^ 3/7 assay. *p < 0.05; n.s.: not significant.

To investigate whether the synergistic anti-tumor effect of pemetrexed + cSBL was mediated by apoptosis, we analyzed activated caspase-3 levels. Western blot analysis demonstrated that all of the treatments increased activated caspase-3 levels (Figure 4A). Caspase-Glo^™^ 3/7 assays indicated pemetrexed and cisplatin did not induce caspase-3 activation. In contrast, a significant increase in activated caspase-3 was observed in cells treated with cSBL alone or with any of the three combination treatments (Figure 4B). There were no significant differences in caspase-3 activity between cells treated with cSBL alone or the combination treatments.

### Pemetrexed and cisplatin inhibit cell proliferation, but cSBL has a cytotoxic effect in H28 cells

**Figure 5:**
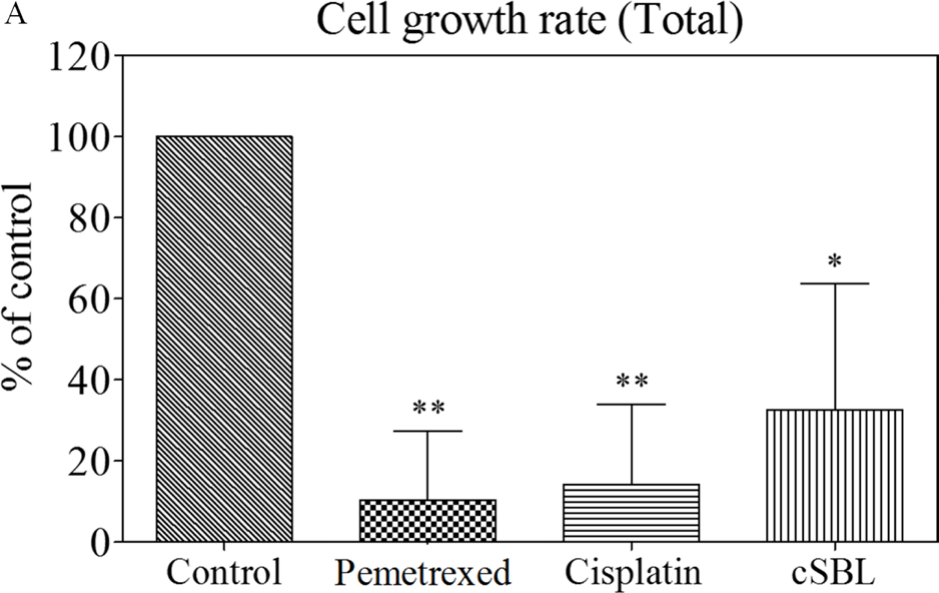

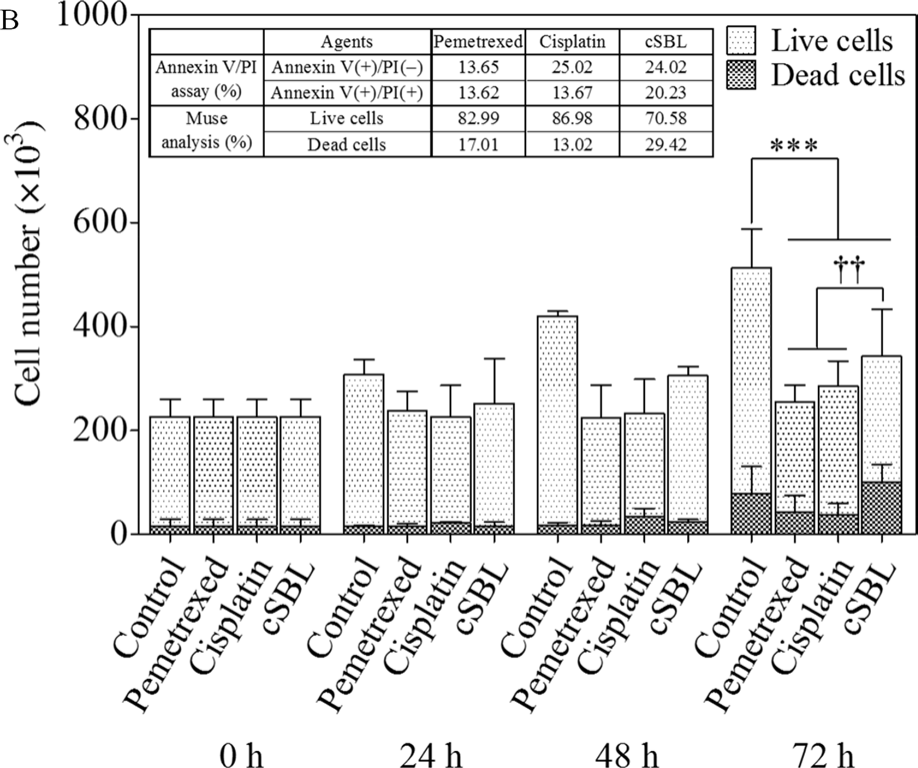
Pemetrexed and cisplatin inhibit proliferation, whereas cSBL has a cytotoxic effect in H28 cells Cells were treated with pemetrexed (20 μM), cisplatin (40 μM), or cSBL (1 μM) for 0–72 h. The number of cells was estimated using a Muse^™^ Count & Viability Kit. (**A**) Cell growth rates were calculated as the ratio of the cell number at 72 h to the cell number at 0 h and presented as a fraction of the controls. (**B**) The number of live and dead cells every 24 h is shown. Statistically significant differences in the live cell number at 72 h were observed in the treatment groups compared to the control (***). Statistically significant differences in the dead cell number at 72 h were observed in cSBL-treated cells compared to cisplatin- or pemetrexed-treated cells (^††^). *p < 0.05, **p < 0.01, ***p < 0.001, ^††^*p* < 0.01.

Because apoptosis was not upregulated with the addition of pemetrexed to cSBL, we investigated the mechanism by which pemetrexed and cSBL inhibited mesothelioma cell viability. Previous reports have indicated that the anti-tumor effect of both pemetrexed and cisplatin is mediated by the induction of apoptosis in response to cell cycle arrest [30–35]. Therefore, we analyzed the effect of pemetrexed and cisplatin on cell proliferation. The total number of cells decreased by 10.4%, 14.2%, and 32.7% in cells treated with pemetrexed, cisplatin, and cSBL, respectively, for 72 h compared to control (PBS-treated) cells (Figure 5A). The ratio of annexin V- and propidium iodide (PI)-positive cells indicated that the number of dead cells barely increased in response to pemetrexed- or cisplatin treatment, whereas the number of dead cells significantly increased with cSBL treatment (Figure 5B).

### The anti-tumor activity of pemetrexed + cSBL is mediated by cytostatic and cytotoxic effects

**Figure 6:**
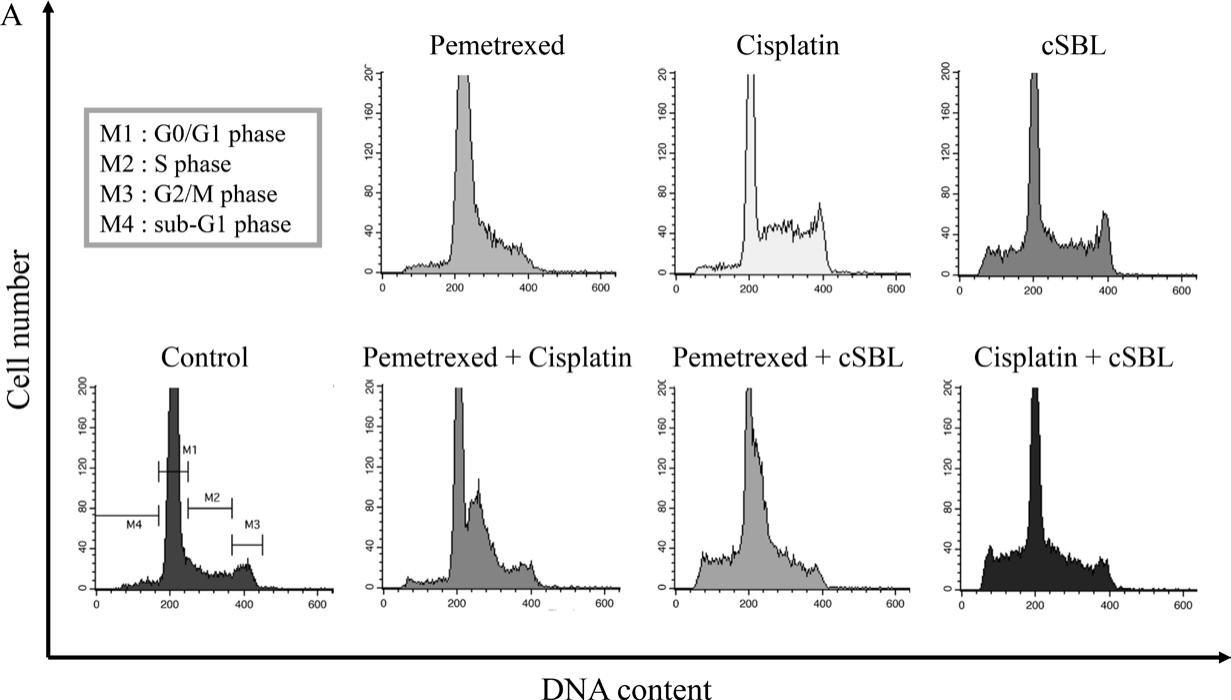

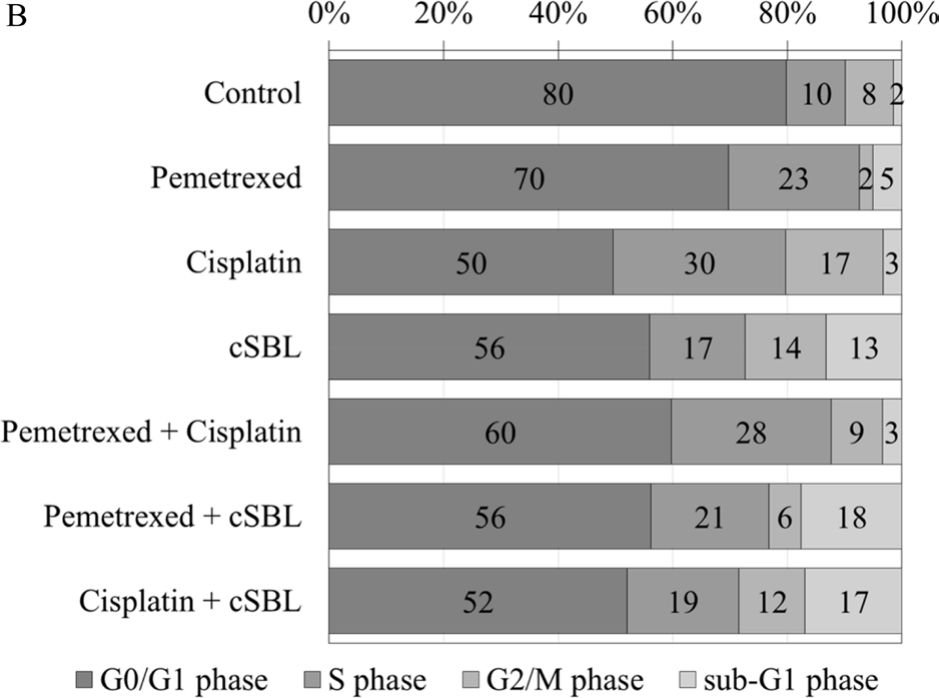

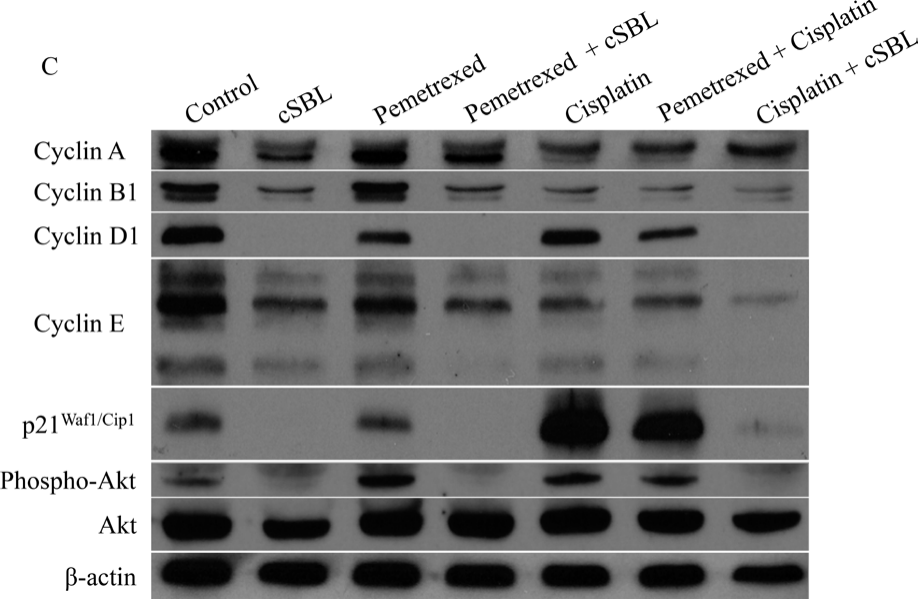
Pemetrexed, cisplatin, and cSBL, either alone or in combination, alter cell cycle dynamics in H28 cells Cells were treated with pemetrexed (20 μM), cisplatin (40 μM), or cSBL (1 μM) for 72 h. (**A**, **B**) Flow cytometry analysis of cell cycle progression in H28 cell lines after 72 h of treatment. (**C**) Western blot analysis of cyclin (A, B1, D1, and E), p21^Waf1/Cip1^, pan-Akt, and phospho-Akt levels.

Cell viability assays suggested that pemetrexed and cisplatin inhibited proliferation. Therefore, we analyzed cell cycle progression in H28 cells treated with these agents. Flow cytometry analysis revealed that pemetrexed and cisplatin induced cell cycle arrest in S phase and the S or G2 phase, respectively. In contrast, cSBL had a minimal effect on cell cycle progression. However, it promoted a significant increase in the number of cells in the sub-G1 phase, indicative of apoptosis. DNA histograms of cells treated with the combination treatments resembled the histograms of cells treated with the individual agents (pemetrexed + cisplatin: S phase arrest, pemetrexed + cSBL: S phase arrest and sub-G1 increment, and cisplatin + cSBL: S or G2 phase arrest and sub-G1 increment) (Figure 6A and 6B). To investigate the molecular mechanisms underlying these effects, we assessed the levels of proteins that regulate cell cycle progression (cyclin, p21^Waf1/Cip1^, and Akt) by western blotting. The levels of cyclin A and p21^Waf1/Cip1^ were unchanged in cells treated with pemetrexed alone, while the levels of phosphorylated Akt significantly increased. Cyclin A and B1 levels significantly decreased in cisplatin-treated cells, while p21^Waf1/Cip1^ levels increased. In contrast, cyclin A, B1, D1, and E levels, and as well as p21^Waf1/Cip1^ and phosphorylated Akt levels, significantly decreased in cSBL-treated cells. The levels of cyclin A and B1 decreased, whereas p21^Waf1/Cip1^ levels increased in cells treated with pemetrexed + cisplatin. Pemetrexed + cSBL, and cisplatin + cSBL, significantly decreased cyclin B1, p21^Waf1/Cip1^, and phosphorylated Akt levels, similar to those observed in cells treated with cSBL alone, whereas pemetrexed + cSBL had the same effect on cyclin A levels as treatment with pemetrexed alone (Figure 6C).

## DISCUSSION

Mesothelioma is categorized as one of three histological subtypes: epithelioid, biphasic, or sarcomatoid [1, 36]. Previous studies have demonstrated that the epithelioid subtype is associated with more favorable survival outcomes compared to non-epithelioid subtypes [1, 37, 38]. We found that cSBL had strong cytotoxic effects in a broader range of mesothelioma cell types including pemetrexed- or cisplatin-resistant cells. We previously demonstrated that cSBL selectively bound to 20 human and animal cancer cell lines but not to 10 normal cell lines [15]. A comprehensive analysis of cSBL cytotoxicity and cancer selectivity was performed previously [39, 40]. We found that cSBL preferentially binds and internalizes into cancer cells compared to normal cells, and exerts cytotoxic effects through its RNase activity [16]. Internalization-defective P-388 mutant (RC-150) cells, which are cSBL-resistant, can bind cSBL but show no cytotoxicity [41]. Therefore, we hypothesize that the lack of specific internalization of cSBL could make normal cells non-sensitive.

Although the effects of cSBL in combination with other agents have been investigated in various cancer cell lines [21, 25, 27], there have been no reports describing the effects of cSBL in combination with pemetrexed or cisplatin. We have demonstrated that the synergistic effect of pemetrexed + cSBL is comparable to that of pemetrexed + cisplatin (Figure 2A and 2B). Treatment with pemetrexed + cSBL could decrease the risk of dose-dependent adverse effects associated with pemetrexed and/or the development of pemetrexed resistance. The synergistic effect of pemetrexed + cSBL is not mediated by increased apoptosis (Figures 3, 4). Apoptosis is commonly observed in cancer cells treated with relatively high concentrations of anti-cancer agents, whereas a cytostatic effect (i.e. transient growth arrest) is typically observed with relatively low concentrations [42]. We found that the percentages of annexin V/PI double-positive cells (Figure 3) and dead cells in the Muse^™^ analysis (Figure 5) of pemetrexed- or cisplatin-treated cells were comparable and relatively low (13–17%). Cell cycle analysis (Figure 6) indicated that pemetrexed and cisplatin exert cytostatic effects, whereas cSBL exerts cytotoxic effects. These data suggest that pemetrexed- and cisplatin-treated cells proceed to an early apoptotic stage, but that cSBL is required for completion of apoptosis. The differential effects of each agent on cell cycle proteins suggest that the molecular mechanisms underlying cell cycle arrest are dependent on the cell type and the specific treatment. Pemetrexed reportedly induces S-phase arrest in A549 lung cancer cells by prolonging Akt activation, thereby sustaining activation of CDK2/cyclin A kinase [31]. In our study, pemetrexed treatment did not significantly alter the levels of cyclin A. However, pemetrexed induced an increase in phosphorylated Akt levels and arrested cells cycle in S-phase (Figure 6). The levels of the cyclins evaluated as well as p21^Waf1/Cip1^ levels significantly decreased in cSBL-treated cells. Since cSBL inhibits RNA translation through degradation, short-lived proteins such as cyclins are likely to be sensitive to cSBL treatment. Interestingly, in cells treated with pemetrexed + cSBL, although the levels of cyclin B1, D1, and E, p21^Waf1/Cip1^, and phosphorylated Akt significantly decreased to levels similar to those observed in cells treated with cSBL alone, cyclin A levels did not significantly change. Although cSBL may inhibit cyclin A translation, pemetrexed might stabilize cyclin A levels and enhance CDK2/cyclin A kinase activity, thereby inducing S-phase arrest and/or apoptosis when used in combination with cSBL [43–45]. The strong synergism of pemetrexed + cSBL is mediated by the cytostatic action of pemetrexed triggered by sustained CDK2/cyclin A activation, and the proapoptotic effect of cSBL.

**Figure 7:**
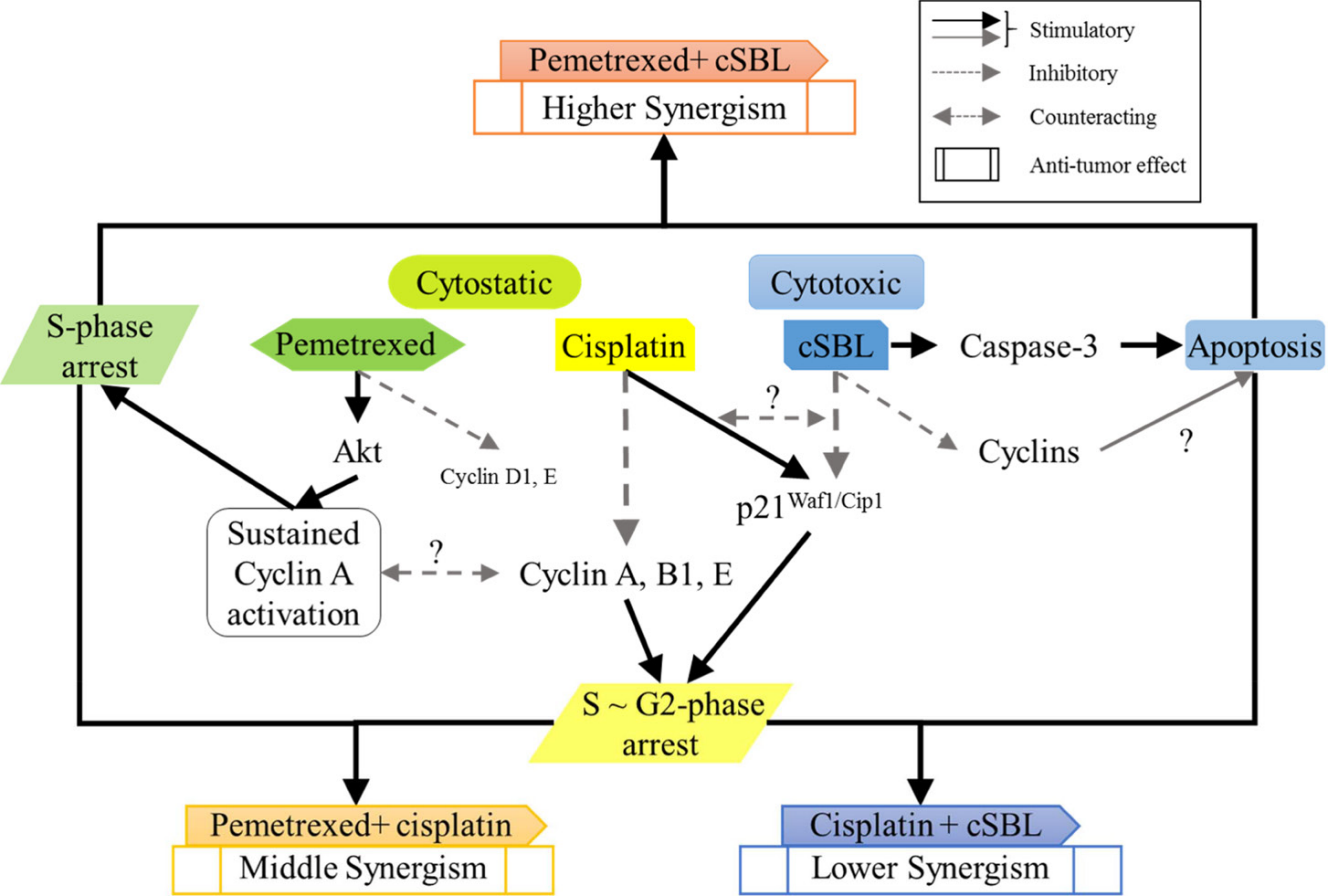
Schematic representation of the molecular mechanisms underlying the synergistic effects of each combination treatment in H28 cells

The levels of p21^Waf1/Cip1^ were differentially affected by each treatment. The tumor suppressor p21^Waf1/Cip1^ arrests cell cycle progression by inhibiting the function of cyclin-CDK complexes or DNA polymerase [46–48]. Overexpression of p21^Waf1/Cip1^ induces cell cycle arrest in G1-, G2- [49], or S-phase [50, 51]. Pemetrexed and cisplatin induce cell cycle arrest by increasing p21^Waf1/Cip1^ levels [30, 52]. As a single treatment, pemetrexed did not change p21^Waf1/Cip1^ levels. However, cisplatin and cSBL significantly increased and decreased p21^Waf1/Cip1^ levels, respectively. Of the combination treatments evaluated, only pemetrexed + cisplatin substantially increased p21^Waf1/Cip1^ levels. Therefore, p21^Waf1/Cip1^ may be important for cisplatin-induced cell cycle arrest in H28 cells. Indeed, p21^Waf1/Cip1^ induced apoptosis in MSTO cells treated with a combination of cisplatin and piroxicam. Silencing p21^Waf1/Cip1^ inhibited this effect [53]. Because cisplatin strongly increased p21^Waf1/Cip1^ in H28 cells (Figure 6), we propose that the decrease of p21^Waf1/Cip1^ levels in cSBL-treated cells might explain the lack of synergy between cisplatin and cSBL. Lazzarini *et al*. reported that shRNA-mediated inhibition of p21^Waf1/Cip1^ enhanced the anti-tumor effects of DNA-damaging agents such as doxorubicin, etoposide, and CPT11 in H28 and H2052 cells [54]. Inoue *et al*. reported that sorafenib downregulated p21^Waf1/Cip1^ levels and promoted cell death in renal cell carcinoma and hepatocellular carcinoma when used in combination with DNA-damaging agents such as paclitaxel or doxorubicin [55]. Therefore, cSBL + DNA-damaging agents (with the exception of cisplatin) might be an effective therapeutic strategy for mesothelioma. The proposed mechanisms of action of the combination treatments are shown in Figure 7.

In conclusion, cSBL exhibits a potent anti-tumor effect in multiple mesothelioma cell lines due to its cytotoxic activity and high selectivity for cancer cells compared to either pemetrexed or cisplatin. Pemetrexed + cSBL exhibited a strong synergistic effect that was comparable or even superior to the standard regimen of pemetrexed + cisplatin. We propose that the synergistic effect results from the combination of the cytostatic effect of pemetrexed and the cytotoxic effect of cSBL. Therefore, cSBL has therapeutic potential for mesothelioma.

## MATERIALS AND METHODS

### Cell culture

The H28, H2452, and MSTO mesothelioma cell lines and immortalized, non-malignant MeT-5A mesothelial cell line were purchased from American Type Cell Culture Collection (Manassas, VA, USA). The MESO-1 and MESO-4 mesothelioma cell lines were obtained from Riken Cell Bank (Tsukuba, Japan). H28, H2452, MSTO, MESO-1, and MESO-4 cells were cultured in RPMI-1640 medium supplemented with 10% fetal bovine serum (FBS). MeT-5A cells were cultured in Medium 199 with Earle's balanced salt solution (75 mM L-Gln and 1.25 g/L sodium bicarbonate) supplemented with 3.3 nM epidermal growth factor (EGF), 400 nM hydrocortisone, 870 nM insulin, 20 mM HEPES, and 10% FBS. All cells were cultured with 100 U/mL penicillin and 100 μg/mL streptomycin at 37°C in a 95% air and 5% CO_2_ atmosphere.

### Reagents

cSBL was isolated using sequential chromatography with Sephadex G75, DEAE-cellulose, hydroxyapatite, and SP-Sepharose as previously described [15]. Pemetrexed disodium heptahydrate was purchased from LC Laboratories (Woburn, MA, USA). Cisplatin was purchased from WAKO Pure Chemical Industries, Ltd. (Osaka, Japan). The caspase-3 polyclonal antibody and cyclin B1 (D5C10), cyclin D1 (92G2), p21^Waf1/Cip1^ (12D1), Akt (pan) (11E7), and phospho-Akt (Ser473) rabbit monoclonal antibodies were purchased from Cell Signaling Technology (Beverly, MA, USA). The cyclin A rabbit polyclonal antibody (H-432) and cyclin E mouse monoclonal antibody (HE12) were purchased from Santa Cruz Biotechnology Inc. (CA, USA). The β-actin antibody from Sigma-Aldrich (Tokyo, Japan) and horseradish peroxidase (HRP)-conjugated anti-mouse IgG were purchased from Zymed (South San Francisco, CA, USA). HRP-conjugated anti-rabbit IgG was purchased from Cedarlane (Hornby, Ontario, Canada), and the Caspase-Glo^™^ 3/7 Assay was purchased from Promega (Madison, WI, USA).

### Cell viability assays

Cell viability was determined using the WST-8 assay. Cells (5 × 10^4^ cells/mL) cultured in 96-well plates (100 μL/well) were treated with various concentration of pemetrexed, cisplatin, or cSBL for 72 h. The cells were incubated with Cell Count Reagent SF (Nacalai Tesque Inc., Kyoto, Japan) at 37°C in a 5% CO_2_ atmosphere for 1–4 h. The absorbance of the resulting product at 450 nm was measured and the background absorbance at 650 nm subtracted. The IC_50_ was calculated using GraphPad Prism 5.0 (San Diego, CA, USA). Experiments were conducted in triplicate.

### Drug combination studies

The effect of combination treatment on cell viability was assessed using WST-8 assays. The concentration of the individual components was based on IC_50_ values obtained in the single treatment experiments. CI values were calculated using the CompuSyn software (ComboSyn Inc., Paramus, NJ, USA) as previously described [56]. CI values < 1 indicated a synergistic effect and CI values > 1 indicated an antagonistic effect.

### Annexin V staining and PI incorporation assays

To evaluate apoptosis, we evaluated annexin V binding and PI incorporation using a MEBCYTO apoptosis kit (MBL, Nagoya, Japan) according to the manufacturer's instructions. Cells (5 × 10^4^ cells/mL) cultured in 12-well plates (1 mL/well) were treated with pemetrexed (20 μM), cisplatin (40 μM), or cSBL (1 μM). Fluorescence intensity was detected using a FACScalibur flow cytometer, and the data analyzed using the CELLQuest software (BD Biosciences, Franklin Lakes, NJ, USA).

### Detection of caspase-3 activity

The level of activated caspase-3 was analyzed using western blot assays with an antibody against cleaved (activated) caspase-3. Cells (5 × 10^4^ cells/mL) cultured in 6-well plates (2 mL/well) were treated with pemetrexed (20 μM), cisplatin (40 μM), or cSBL (1 μM) for 72 h. Whole cell lysates were prepared using extraction buffer (150 mM NaCl, 10 mM Tris-HCl [pH 7.4], 5 mM EDTA, 1% Nonidet P-40, 0.1% sodium deoxycholate, and 0.1% sodium dodecyl sulfate) supplemented with 1 tablet/10 mL cOmplete, Mini, EDTA-free Protease Inhibitor Cocktail (Roche Applied Science, Indianapolis, IN, USA) and 1 tablet/10 mL PhosSTOP phosphatase inhibitor (Roche Applied Science). Soluble proteins were collected and protein concentration measured using a BCA Protein Assay Kit (Thermo Fisher Scientific, Waltham, MA, USA) according to the manufacturer's instructions. The proteins were separated using SDS-PAGE and transferred to Immobilon-P Transfer Membranes (Merck Millipore, Billerica, MA, USA). Membranes were sequentially incubated with primary and secondary antibodies diluted in Can Get Signal (Toyobo CO., LTD., Osaka, Japan). The protein bands were detected using ECL Prime Western Blotting Detection Reagent (GE Healthcare, Little Chalfont, UK).

Caspase-3 enzymatic activity was measured using a Caspase-Glo^™^ Assay. Cells (5 × 10^4^ cells/mL) cultured in white 96-well plates (25 μL/well) were treated with pemetrexed (20 μM), cisplatin (40 μM), or cSBL (1 μM) for 72 h in triplicate. Caspase-Glo^™^ Reagent (25 μL) was added to each well and the contents of the wells mixed using a plate shaker for 30 seconds. The cells were incubated at 37°C in a 5% CO_2_ atmosphere for 1 h. The luminescence in each well was measured using GloMax^™^ Multi Detection System (Promega, Madison, WI, USA).

### Cell proliferation assays

Cell proliferation was quantified using a Muse^™^ Count & Viability Kit (Merck Millipore, Billerica, MA, USA). Cells (5 × 10^4^ cells/mL) cultured in 24-well plates (500 μL/well) were treated with pemetrexed (20 μM), cisplatin (40 μM), or cSBL (1 μM) for 0–72 h. The cells were collected at 24 h intervals and combined with Muse^™^ Count & Viability Reagent in which both viable and non-viable cells are differentially stained based on their permeability to the DNA-binding dyes (cells:reagent = 1:9). The total number of viable or dead cells was counted using a Muse^™^ Cell Analyzer (Merck Millipore).

### Cell cycle analysis

Changes in cell cycle progression induced by 72 h of treatment with pemetrexed (20 μM), cisplatin (40 μM), or cSBL (1 μM) were evaluated using a CycleTEST^™^ Plus DNA Reagent Kit (BD Biosciences) according to the manufacturer's instructions. Cells (5 × 10^4^ cells/mL) were cultured in 12-well plates (1 mL/well). Fluorescence intensity was detected using a FACScalibur flow cytometer and the data analyzed using the CELLQuest software (BD Biosciences). Cell cycle progression was analyzed using Flowing Software 2 (Perttu Terho, Turku Centre for Biotechnology, Finland). The levels of cell cycle regulators (cyclin A, B1, D1, E, and p21^Waf1/Cip1^), pan-Akt, and phospho-Akt were evaluated by western blot analysis.

### Statistical analysis

The results from at least three independent experiments performed in triplicate are expressed as the mean ± standard deviation (SD). Statistical analyses were conducted using GraphPad Prism 5.0 and comparisons made using one-way analysis of variance (ANOVA) followed by Bonferroni post-hoc tests.
